# Assessment of hand hygiene facilities and staff compliance in a large tertiary health care facility in northern Nigeria: a cross sectional study

**DOI:** 10.1186/s13756-020-0693-1

**Published:** 2020-02-11

**Authors:** Kenneth I. Onyedibe, Nathan Y. Shehu, Daniela Pires, Samson E. Isa, Mark O. Okolo, Simji S. Gomerep, Comfort Ibrahim, Sunday J. Igbanugo, Rachel U. Odesanya, Adebola Olayinka, Daniel Z. Egah, Didier Pittet

**Affiliations:** 1 Department of Medical Microbiology, University of Jos, Jos, Nigeria; 2 Infectious Diseases Unit, Department of Medicine, University of Jos, Jos, Nigeria; 3 Infection Control Programme and WHO Collaborating Centre on Patient Safety - Infection Control & Improving Practices, University of Geneva Hospitals and Faculty of Medicine, Geneva, Switzerland; 4 Department of Infectious Diseases, Centro Hospitalar Lisboa Norte and Faculdade de Medicina da Universidade de Lisboa, Lisbon, Portugal; 5 Department of Nursing Services, Jos University Teaching Hospital, Jos, Nigeria; 6 Department of Pharmacy, Jos University Teaching Hospital, Jos, Nigeria; 7 Department of Medical Microbiology, Ahmadu Bello University, Zaria, Nigeria

**Keywords:** Hand hygiene, Facilities assessment, Compliance, Developing countries, Alcohol-based handrub, Hand sanitizer, WHO multimodal strategy

## Abstract

**Background:**

The burden of healthcare-associated infection (HAI) is 2 to 18 times higher in developing countries. However, few data are available regarding infection prevention and control (IPC) process indicators in these countries. We evaluated hand hygiene (HH) facilities and compliance amongst healthcare workers (HCW) in a 600-bed healthcare facility in Northcentral Nigeria providing tertiary care service for a catchment population of about 20 million.

**Methods:**

An in-house facility assessment tool and the World Health Organization (WHO) direct observation method were used to assess the HH facilities and compliance, respectively. Factors associated with good compliance were determined by multivariate analysis.

**Results:**

The facility survey was carried out in all 46 clinical units of the hospital. 72% of the units had no poster or written policy on HH; 87% did not have alcohol-based hand rubs; 98% had at least one handwash sink; 28% had flowing tap water all day while 72% utilized cup and bucket; and 58% had no hand drying facilities. A total of 406 HH opportunities were observed among 175 HCWs. The overall compliance was 31%, ranging from 18% among ward attendants to 82% among medical students. Based on WHO “5 moments” for HH, average compliance was 21% before patient contact, 23% before aseptic procedure, 63% after body fluid exposure risk, 41% after patient contact and 40% after contact with patients’ surrounding. Being a medical student was independently associated with high HH compliance, adjusted odds ratio: 13.87 (1.70–112.88).

**Conclusions:**

Availability of HH facilities and HCW compliance in a large tertiary hospital in Nigeria is poor. Our findings confirm that HCWs seem more sensitized to their risk of exposure to potential pathogens than to the prevention of HAI cross-transmission. Inadequate HH facilities probably contributed to the poor compliance. Specific measures such as improved facilities, training and monitoring are needed to improve HH compliance.

## Background

Improving hand hygiene (HH) reduces the transmission of healthcare-associated pathogens and healthcare-associated infections (HAI) [1]. In the United Sates, it has been estimated that HAI incidence ranges from 1.7 to 23.6 per 100 admitted patients accounting for direct annual hospital costs of 28.4 to 33.8 billion U.S. dollars [2] and for approximately 80,000 deaths per year [3]. More importantly, HAI burden is estimated to be up to 18 times higher in developing countries when compared with developed countries [4]. Yet, HAI are frequently preventable through infection prevention and control (IPC) measures, with HH as key activity [5, 6]. This reality has led organizations such as the Joint Commission, the World Health Organization (WHO), Centers for Disease Control and Prevention (CDC) and European Center for Disease Prevention (ECDC) to recommend HH practices and interventions in all healthcare facilities worldwide [ 1, 7, 8]. There are two possible ways of performing HH: hand washing with soap and water or hand rubbing with alcohol-based hand rubs (ABHR) [1, 5–8]. The WHO itemized five key moments when healthcare workers (HCWs) should practice HH: these are before patient contact, before an aseptic procedure, after bodily fluid exposure risk, after patient contact, and after contact with patient surroundings. Monitoring HCWs compliance with HH practices is vital for evaluating whether HH interventions are successful. WHO recommends using a validated methodology for training observers to directly monitor HH using “My five moments for hand hygiene” [1, 9, 10].

In Nigeria, the overall prevalence of HCAIs ranges between 2.6 and 30.9%, while the cumulative incidence among surgical patients ranges from 5.7 to 48% [11]. Many fatal infections, such as the nosocomial transmission of endemic Lassa fever in Nigeria and the West African outbreak of Ebola which occurred also in Nigeria might have been curtailed much earlier if IPC measures and HH facilities were in place and healthcare workers (HCWs) adhered to standard HH and other IPC practices.

As many factors including inadequate IPC facilities contribute to this problem, there is therefore need to assess IPC facility availability and functionality in Nigerian hospitals. We conducted a survey of availability of HH facilities and HH compliance at a tertiary Nigerian hospital while also determining factors associated with compliance.

## Methods

### Study design and setting

The study site is a 600-bed capacity tertiary health care center in Plateau state, North Central Nigeria, serving a population of around 20 million which includes that of the neighboring 8 states as a major referral hospital. Plateau state is located within an area of 26,899 km^2^. It is located between latitude 80*24 N and longitude80*32 and 100*38 east. Majority of the inhabitants are either farmers or civil servants. Health care delivery is structured into three tiers: primary, secondary and tertiary health care delivery services and healthcare financing is largely out-of-pocket. Primary and secondary healthcare facilities in the neighboring states refer patients to the study site. The study site has 46 wards and units including an intensive care unit (ICU) and the Special care baby unit. Most wards are designed as open halls with an average capacity of 22- beds. There are a few single-bed private rooms for patients that can afford the fee. The hospital has about 20,000 annual outpatient medical consultations and over 10,000 annual admissions. It has 17 clinical departments with specialists: anaesthesia, community medicine, chemical pathology, family medicine, haematology and blood transfusion, histopathology, medical microbiology, obstetrics & gynaecology, ophthalmology, orthopaedics & trauma, otorhinolaryngology (ENT), psychiatry, radiology and various sub-specialist surgeons and internists.

There were an estimated 150 nurses, 90 doctors, 130 medical students, 55 nursing students, and 150 attendants that were actively working at the relevant units when the assessment of HH facilities and compliance were carried out.

Three months prior to this assessment of hand hygiene compliance, HH sensitization was done by the hospital’s infectious diseases physician and medical microbiologist for nurses, doctors, pharmacists and hospital attendants with only about 30% attendance of HCWs. This consisted of HH lectures followed by brief question and answer sessions. Before this, there had not been a comprehensive HH campaign according to WHO multimodal strategy in the hospital.

### HH facilities assessment

A one-day (20/11/2013) point prevalence survey of available HH facilities within the tertiary health facility was carried out. This was by the use of a modified Infection Control Self-Assessment Tool [12] that captures multiple parameters and easily adaptable to our setting. Trained study members systematically used the check list to capture relevant data from all clinical wards and other units of the hospital where patients have direct access. Data collected were on availability of tools/items related to water supply and HH facilities; such as presence of ABHRs; presence and location of sinks, functionality of sinks; presence of hand dryers or disposable towels; availability of water, soap/handwash and availability of written HH policies, job aids and/or posters. Data were collected by direct observation and HCWs interviews in the various hospital units.

### HH compliance

Phase two was an observational study of HCWs compliance to HH performed from January 2014 to March 2014. This was the first audit of HH compliance ever conducted in the hospital. Members of the Clinical Infection Research Group (CIRG) of the hospital were trained on the WHO direct observation method. This training was conducted by experienced infectious disease physicians and medical microbiologists using a simulation method. We evaluated the level of compliance across different wards and units and among different categories of HCWs. The HH observations were conducted in the 6 bed-ICU, Medical, Surgical, Pediatrics, Obstetrics and Gynaecology wards, Emergency wards, laboratories and the Pharmacy unit. HH compliance monitoring was conducted and evaluated using the direct observation technique described in the WHO Hand Hygiene Technical Reference Manual (HHTRM) [ 13].

### Sample size for compliance assessment

The sample size was calculated only for HH compliance assessment. According to the Public health Ontario HH compliance and observation analysis and observation standards, it is estimated that 56 observation sessions are needed to be collected in order to obtain reliable estimates of compliance in a 100-bed capacity health institution [14]. This translates to a minimum of 336 observations in a 600-bed facility. We observed 406 HH opportunities in the 600-bed institution. Overall compliance was determined by dividing the number of observed hand hygiene actions performed when an opportunity occurs, by the total number of opportunities. Hand hygiene compliance of > 50% was considered good compliance [15, 16].

### Data collection and analysis

Data collected by the trained observers were entered into the standard WHO observation proforma [13]. Observed compliance rates conducted in the different wards and amongst different HCW categories were analyzed using Epi-Info version 3.5.1 (CDC, Atlanta, Georgia). Chi square test was used to compare differences in proportions and *P* values of < 0.05 were considered statistically significant. We used bivariate analysis to identify factors associated with good compliance. Variables with *p* values < 0.25 were subjected to a multiple logistic regression analysis model [17, 18].

### Ethical consideration

Ethical clearance for the study was obtained from the Research and Ethics Committee of the University Teaching Hospital. All data were securely and confidentially kept.

## Results

For the overall facility assessment, all 46 hospital wards and clinical units were assessed and this is shown in Table 1.

**Table 1:** Distribution of hand hygiene facilities at a 600 bed-tertiary care hospital, in Jos, Northern Nigeria

Units/wards of the hospital	Frequency(n)	No poster/ written policy on HH^a^	No ABHR^a^	Position and type of ABHR	Hand washing station	Dominant Position (average distance #) of HWS^a^	Water usually available all day	Hand operated tap in wash station	Use of bar soap	Availability of soap always	Use of ladle/cup and bucket	Multiple-use cloth-towel for hand drying	Automated hand dryers
Out-patient clinics	9	7(78)	7(78)	0	9(100)	Adj. room(8 m)	0 (0)	9(100)	7 (78)	3 (33)	7 (78)	5 (56)	0 (0)
Laboratories	5	4(80)	3 (60)	pocket	5 (100)	WB (3 m)	1(20)	5 (100)	3 (60)	1 (20)	1 (20)	1 (20)	0 (0)
Wards	21	15(71)	20 (95)	NS, pocket	21 (100)	NS (8 m)	8 (38)	21 (100)	20 (95)	15 (71)	15 (71)	2 (10)	0 (0)
Emergency wards	3	3(100)	3(100)	nil	3(100)	Adj.room (5 m)	0 (0)	3(100)	3(100)	1 (33)	1(33)	3(100)	0 (0)
ICU	1	0(0)	0 (0)	NS, pocket	1(100)	Patient bedside(1 m)	1 (100)	1(100)	1(100)	1(100)	1(100)	0 (0)	0 (0)
Operating theatre	1	1(100)	1(100)	nil	1(100)	Lobby(3 m)	1 (100)	1(100)	1(100)	1(100)	1(100)	0 (0)	0 (0)
Delivery suite	1	0 (0)	1(100)	nil	1(100)	Lobby(3 m)	1 (100)	1(100)	1(100)	1(100)	1(100)	0 (0)	0 (0)
Mortuary	1	0 (0)	1(100)	nil	1(100)	Lobby(3 m)	0 (0)	1(100)	1(100)	0 (0)	1(100)	1(100)	0 (0)
Dialysis unit	1	0 (0)	1(100)	nil	1(100)	Lobby(3 m)	1 (100)	1(100)	1(100)	0 (0)	0 (0)	0 (0)	0 (0)
ECG unit	1	1(100)	1(100)	nil	0 (0)	nil	0 (0)	0 (0)	0(0)	0 (0)	1(100)	0 (0)	0 (0)
Physiotherapy unit	1	1(100)	1(100)	nil	1(100)	Lobby (4 m)	0(0)	1(100)	1(100)	1(100)	1(100)	1(100)	0(0)
Endoscopy suite	1	1(100)	1(100)	nil	1(100)	suite(3 m)	0(0)	1(100)	1(100)	0(0)	1(100)	1(100)	0(0)
Total	46	33(72)	40(87)		45(98)		13(28)	45(98)	40(87)	24(52)	31(67)	14(30)	0(0)

^a^*HH* Hand hygiene, *ABHR* Alcohol based hand rub, *HWS* hand wash station, # Estimated distance, *NS* Nurses Station, *WB* Work bench

**Fig. 1:**
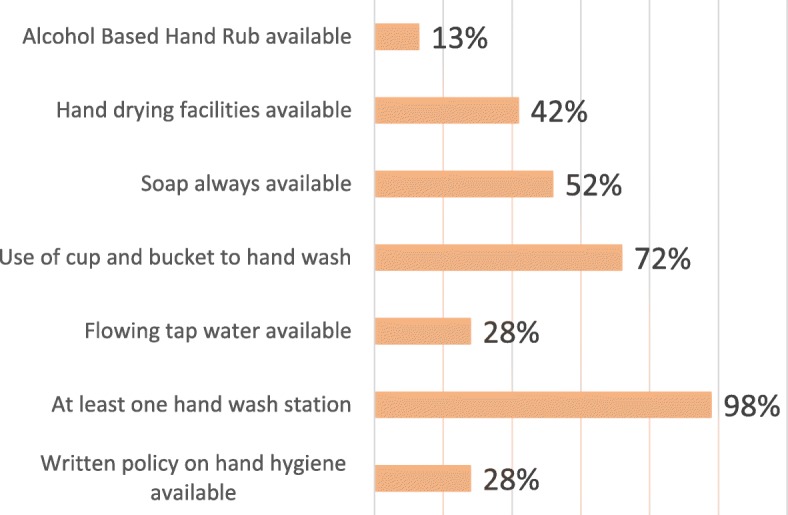
Proportion of hospital units with available hand hygiene facilities

A total of 40 (87%) units did not have ABHRs. Thirty-three units (72%) had no poster or written policy on hand hygiene. In the 6 units (13%) where ABHR was available, all were personal pocket-size ABHRs. Forty five (98%) units had at least one hand washing station. Of these, only 13 (28%) had all day availability of piped water and 32 (72%) handwashing stations utilized a ladle or cup and bucket (Fig. 1). All sinks were hand operated located by the nurses’ station in the open ward and 40 units (87%) routinely used bar soap, the remaining 13% either used liquid handwash or just water depending on availability. However, soap or liquid handwash was always available only in 24 units (52%). Nineteen (42%) of the units used multiple-use cloth-towel for hand drying which were changed daily on the average. There were no automated hand dryers or paper towels in any of the units.

**Fig. 2:**
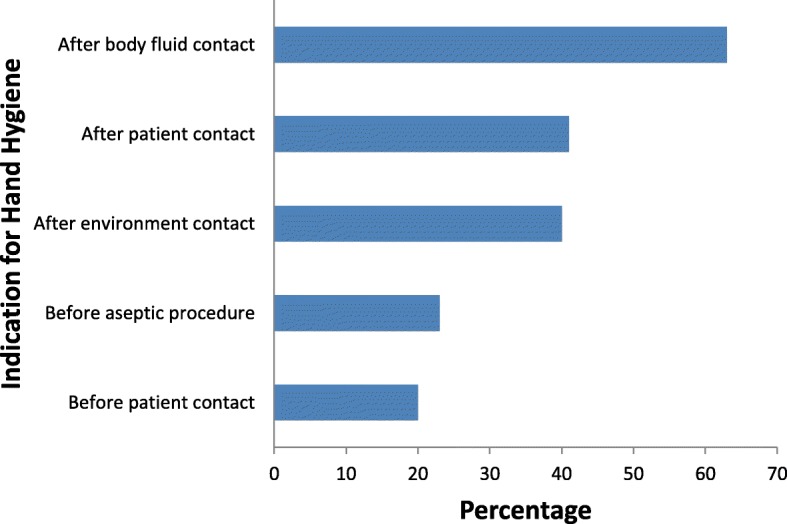
Compliance with the WHO “5 moments of hand hygiene”

**Fig. 3:**
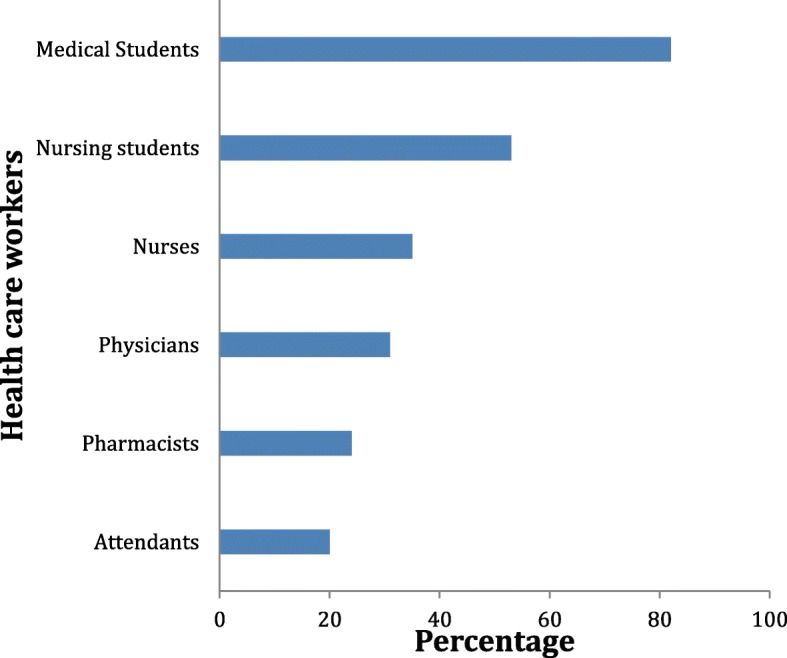
Compliance with hand hygiene by healthcare worker category in a tertiary hospital, Nigeria

A total of 175 HCWs were observed for compliance with HH practices; these were ward attendants (*n* = 54, 30.9%); nurses (42, 24%); medical doctors (27, 15.4%); nursing students (21, 12%); pharmacists (18, 10.3%); and medical students (13, 7.4%). We observed a total of 406 opportunities for HH; overall compliance was 126/406, 31% (95% CI, 27–36). The large majority (103/126, 82%) of the HH actions were performed using handwashing, while only 23/126 (18%) were through the use of ABHRs. Hand hygiene compliance was 105/283, 37% on week-days and 20/90, 22% on weekends, *P* value was 0.017. Compliance to the 5 moments of HH varied according to indications for HH; from a highest of 63% after body fluid exposure risk to 21% before patient contact, 41% after patient contact, 40% after contact with patient environment and 23% before aseptic procedure (Fig. 2). Stratifying by HCW category, compliance ranged from a maximum of 82% among medical students to a minimum of 20% among ward attendants (Fig. 3). Factors associated with hand hygiene compliance are shown in Table 2; as shown, being a medical student was independently associated with good HH compliance (*p* = 0.01), while working as a ward attendant was independently associated with poor compliance (*p* = 0.031).

**Table 2:** Factors associated with good compliance with hand hygiene in Jos, 600 bed-tertiary care hospital, Northern Nigeria

Variables	Univariate OR (95% CI)	*P* value	Multivariate AOR (95% Cl)	*P* value
Week days (Yes/No)	2.66 (1.18–6.36)	0.017	1.70 (0.68–4.23)	0.26
Non ICU (vs ICU)	0.69 (0.24–1.86)	0.45	NA	NA
Physicians^a^	0.92 (0.39–2.12)	0.84	NA	NA
Pharmacists^a^	0.49 (0.15–1.40)	0.18	0.61 (0.18–2.04)	0.42
Nurses^a^	1.31 (0.65–2.65)	0.45	NA	NA
Medical students^a^	18.90 (3.16–417.12)	0.0002	13.87 (1.70–112.88)	0.01
Nursing students^a^	2.81 (1.07–7.91)	0.03	2.27 (0.80–6.40)	0.12
Attendants^a^	0.31 (0.15–0.63)	0.0001	0.42 (0.19–0.93)	0.031

Good compliance with hand hygiene was defined as compliance greater than 50%

*NA* Not applicable (Only variables with *p* values < 0.25 on univariate were put into multivariate regression model), *OR* odds ratio, *AOR* adjusted odds ratio

^a^Compared to other healthcare workers’ categories

## Discussion

Hand hygiene is the most important means of preventing HAI [19]. Over the years, immense efforts have been made at improving HH compliance worldwide. A major challenge to these efforts remains the availability of functional and accessible HH facilities. Our study revealed low access to HH facilities and poor access to today’s key cue for action, HH. ABHR was used in only 13% of the units studied in the hospital, a situation that compels most HCWs to either use soap and water, or forget to wash their hands. This might explain why HCWs in the study performed HH with ABHR in only 18% of the HH opportunities observed. Studies previously demonstrated that access to ABHR, associated with multimodal promotion, was critical to improve HH compliance, in both developed and developing countries healthcare settings [15, 16, 20, 21].

This study also revealed that availability of handwashing stations was high (98%). However, only 28% of the sinks were fully functional with water flowing from their taps all day. This is quite low when compared to reports from a similar study in India with 98% functional and accessible handwashing sinks [22]. Although our study had a low proportion of functional sinks, a similar Nigerian study found an even lower proportion (14.3%) [23]. It appears that healthcare funding challenges, poor maintenance attitude and erratic water supply, play a role in the lack of functional sinks in our study, as previously mentioned by Busari et al. [23] Limited access to HH facilities has been shown to be an important risk factor for poor compliance to HH [15, 16, 20–23]. In the absence of constant water supply, cleanliness of sinks becomes even more questionable and such sinks may themselves become a source for resistant pathogens and both endemic and epidemic HAIs. Studies identified handwashing sinks as a source of highly fatal multidrug-resistant Klebsiella oxytoca and other enterobacteriacea [24, 25]. The cleanliness of sinks and potential carriage of pathogens was not evaluated in the current study and should be considered in future research.

Furthermore, all the taps in our study were hand operated. Similar findings of hand-operated taps were reported by Busari et al. in Nigeria (100%) [23], Devnani et al. in India (99.5%) [22] and Amanzian et al. (93%) in four Mediterranean countries (Egypt, Morocco, Algeria and Tunisia) [26]. There is an increased risk of contamination of hands and subsequent cross-transmission through hand operated sinks [23–26]. Current recommendations encourage the use of elbow-sensor-operated, or automated taps for handwashing [27–29]. Moreover, in a large proportion of the units, cups, bowls or a ladle were used to pour water from a bucket to HCWs hands. This practice is challenging when there is no assistant to pour water on hands, further increasing the risk of recontamination of washed hands due to continued contact with the unwashed ladle or cup which had been touched previously with unclean hands. In addition, reusable cloth towels were in use; there were no disposable towels or automated hand dryers in most units as recommended in current guidelines [1, 27–29]. Reusable towels are another potential source of recontamination of HCWs hands and may contribute to spread of HAIs, as reported by other studies [30]. Cross-transmission by reusable towels can mimic ‘hand-to-hand’ transmission when hands are not washed properly and if the concentration of bacteria on the towel is high enough [31]. The cleanliness of some of the reusable towels, the unavailability of ABHR and the lack of constant water supply could discourage HCWs from performing HH and might have been the case in this study.

Therefore, the overall HH compliance observed in the current study (31%) was not surprising considering the poor availability of HH facilities. Similar compliance rates were reported from China (30%) and Kuwait (33.4%) [32, 33]. Our compliance rate, although lower than acceptable threshold, is higher than previous reports from some other parts of Nigeria (16.7%), Ethiopia (16.5%), Mali (8%) and Indonesia (20%) [20, 34–36]. Higher rates were reported in Saudi Arabia (50.3%), southwest Nigeria (55%) and southeast Nigeria (65.3%) [37–39]. The high compliance rate observed in the Southeastern and Southwestern Nigerian studies may be attributed to the positive effect of the interventional programs mentioned in the studies which included systematic HH training, and the use of HH posters and other reminders in the hospital facilities. These measures have been shown to improve HH compliance [20, 40, 41].

In our study, HH compliance was higher among nurses (36%) than among doctors (31%). This is consistent with most studies in the literature including a meta-analysis [42]. However, compliance among doctors in our study is higher than reported from an earlier study among doctors in Southern Nigeria (16.7%) [43] which may suggest that the knowledge and practice of HH is improving among doctors. The comparatively high compliance rates observed among medical (82%) and nursing (55%) students was rather surprising, and has not been reported in previous studies [38, 42]. This is probably due to less workload for the students, a factor known to positively influence HH compliance. The high compliance amongst students might also be due to close supervision by trainers and to recent curriculum modifications, where IPC and HH have been introduced as a new module taught to the students.

Contrary to what has been described [44], we found no independent association between working in the ICU with lower HH compliance rates. The difference with the current study may be due to the comparatively fewer patients in our 6 bed-ICU and the relatively low number of opportunities observed. Pittet et al. had shown that settings with very high number of HH opportunities (> 60/h of patient care) would have generally poor compliance rates compared to low (0 to 20 HH opportunities) [44, 45]. We also did not find any independent association between working on weekends and poor HH compliance. This may be due to fewer HH observations done on weekends compared to week-days in our study.

Hand hygiene compliance before patient contact was lower (21%) than after patient contact (41%). This trend is similar to that reported in several studies, including a meta-analysis that found lower compliance rates before patient contact (21%) compared to after patient contact (47%) [42]. HCWs are more conscious of acquiring infections from patients and the health care environment [46], and our findings confirm that they tend to be more concerned about protecting themselves from acquiring an infection rather than protecting patients. Shobowale et al. in a study in Nigeria, also, found higher compliance after body fluid exposure of about 60% which is fairly similar to the 63% in our study. However, they found a comparatively higher compliance rate before aseptic technique [38]. This may be due to the higher availability of HH facilities at the areas where aseptic procedures were carried out in their study. In a similar study that found a low HH compliance in Nigeria, the authors suggested that some HCWs assume that wearing gloves reliably replaces HH [47]. We also found a low HH compliance after contact with patient surrounding, this is similar to the study by FitzGerald et al. where HH after contact with patient surrounding appears to be the most commonly missed/neglected moment of HH [48]. These findings were not unexpected as an overall poor knowledge of HH in the study setting was previously reported [49].

## Conclusions

HH facilities were inadequate and HCW compliance to HH was low. HH was performed more after exposure risk to body fluid and patient contact than to other moments and compliance was better during weekdays. Administrative and engineering challenges such as non-availability of piped water, functional handwash stations and poor availability of ABHR may have contributed to the low level of compliance to HH in this study. Measures to improve HH compliance such as implementing the WHO multimodal HH strategy, that includes training, system change with the provision of ABHR and other HH facilities, and monitoring and feedback of hand hygiene compliance, are needed in our setting and similar settings.

## Data Availability

The datasets used during the current study are available from the corresponding authors on request.

## Abbreviations

ABHR: alcohol-based hand rubs
CDC: Centers for Disease Control and Prevention
ECDC: European Center for Disease Prevention
HAI: Healthcare-associated infection
HCW: Healthcare workers
HH: Hand hygiene
HHTRM: Hand Hygiene Technical Reference Manual
ICU: Intensive care unit
IPC: Infection prevention and control
WHO: World Health Organization
